# Effect of Rotating Auditory Scene on Postural Control in Normal Subjects, Patients With Bilateral Vestibulopathy, Unilateral, or Bilateral Cochlear Implants

**DOI:** 10.3389/fneur.2018.00972

**Published:** 2018-11-16

**Authors:** Caroline Guigou, Michel Toupet, Benoit Delemps, Sylvie Heuschen, Serge Aho, Alexis Bozorg Grayeli

**Affiliations:** ^1^Department of Otolaryngology-Head and Neck Surgery, Dijon University Hospital, Dijon, France; ^2^Le2i Research Laboratory, CNRS, UMR-6306, Dijon, France; ^3^Centre d'Explorations Fonctionnelles Otoneurologiques, Paris, France; ^4^Audika Auditory Rehabilitation Center, Dijon, France; ^5^Department of Epidemiology, Dijon University Hospital, Dijon, France

**Keywords:** binaural hearing, stereophony, balance, bilateral vestibulopathy, posturography, multisensory integration

## Abstract

**Objective:** The aim of this study was to investigate the impact of a rotating sound stimulation on the postural performances in normal subjects, patients with bilateral vestibulopathy (BVP), unilateral (UCI), and bilateral (BCI) cochlear implantees.

**Materials and Methods:** Sixty-nine adults were included (32 women and 37 men) in a multicenter prospective study. The group included 37 healthy subjects, 10 BVP, 15 UCI, and 7 BCI patients. The average of age was 47 ± 2.0 (range: 23–82). In addition to a complete audiovestibular work up, a dynamic posturography (Multitest Framiral, Grasse) was conducted in silence and with a rotating cocktail party sound delivered by headphone. The center of pressure excursion surface (COPS), sensory preferences, as well as fractal, diffusion, and wavelet analysis of stabilometry were collected.

**Results:** The rotating sound seemed to influenced balance in all subgroups except in controls. COPS increased with sound in the BVP and BCI groups in closed eyes and sway-referenced condition indicating a destabilizing effect while it decreased in UCI in the same condition suggesting stabilization (*p* < 0.05, linear mixed model corrected for age, *n* = 69). BVP had higher proprioceptive preferences, BCI had higher vestibular and visual preferences, and UCI had only higher vestibular preferences than controls. Sensory preferences were not altered by rotating sound.

**Conclusions:** The rotating sound destabilized BVP and BCI patients with binaural hearing while it stabilized UCI patients with monaural hearing and no sound rotation effect. This difference suggests that binaural auditory cues are exploited in BCI patients for their balance.

## Introduction

The interaction between binaural hearing and space representation has been discussed for several decades. In 1960, Hennebert studied the clinical effects of a rotating sound in healthy subjects and observed a provoked nystagmus which he called “audiokinetic.” This nystagmus had a slow phase parallel to the source movement. Other reported effects were a deviation of the upper limbs during the Romberg test, deviation of vertical writing toward the sound and also neurovegetative reactions (1).

Bats are probably the most performant animals in using their hearing for the representation of their environment (2). These animals can separate auditory cues related to echolocation from those used for communication during their flight (2) and echolocation seems to be quite efficient since it does not add large energetic costs to the aerodynamic power requirements of their flight (3). After several months of training, many blind humans can also develop echolocation using tongue clicks. They use this capacity in daily life to avoid obstacles and to obtain information on the form and the size of surrounding objects (4). Echolocation is a dynamic process and uses the head-related transfer function (4–6). This ability requires stereophony with two equivalently performant ears (7). In blind experts, a separate processing of auditory source-motion and echo-motion in temporal-occipital cortex is observed and fMRI data suggest central reorganization with a possible recruitment of visual cortex (8).

Other observations on postural behavior of normal subjects submitted to static or mobile sound sources support the idea that hearing afferences could have an impact on the gait when other afferences are destroyed or ineffective (9–11). Studies in elderly patients are in line with those in experimental conditions on the role of auditory input in the balance by showing that hearing aids enhance the gait during Romberg test, decrease the risk of falls (12) and improve many aspects of quality of life related to balance (13).

In patients with a cochlear implant (CI), the effect of sound on balance has been rarely reported (14). Many bilaterally deaf patients still receive CI on one side due to its cost (15). Today, based on proven benefits of binaural hearing on global auditory performances, bilateral CI (BCI) is proposed more frequently in developed countries (16). However, BCI does not provide stereophony in all patients due to asymmetries of auditory nerve function, and to the sound coding strategies which reduce or suppress binaural time cues (17). Although the effect of BCI on sound localization has been reported (17, 18), the influence of binaural cues provided by CI on balance performances has not been investigated to our knowledge. It should also be underlined that CI has a potential impact on vestibular integrity and function. Histological lesions in the saccule and semicircular canals as a consequence of CI have been described (19). However, the functional consequences in unilateral cochlear implantees (UCI) appear to be mild (20).

Recent studies on sound-gait interaction provide different and sometimes contradicting results, but they all suggest an effect of the sound on balance performances (21–27). The contradictions are probably related to the experimental protocol, the characteristics of the subjects, the measured parameters and the fact that balance is a dynamic process which uses different sensory inputs changing in hierarchy depending on patients and on situations (28).

In order to better understand the interaction between hearing and vestibular functions, especially in patients with CI, we aimed at investigating the effect of moving sound sources on balance performances by dynamic posturography in healthy subjects, in patients with bilateral vestibulopathy (BVP), and also in UCI and BCI.

## Materials and methods

### Population

Sixty-nine subjects were included in this prospective and multicenter study from September 2015 to February 2016. The population included 37 healthy volunteers, 10 BVP, 15 UCI, and 7 BCI. The group was composed of 32 women and 37 men. The mean age was 47 ± 2.0 years [range: 23–82] in the general population, 38 ± 2.1[23–66] for controls, 63 ± 2.4 [50–74] for BVP, 53 ± 3.7[23–71] for UCI, and 57 ± 9.2[25–82] for BCI. Controls were younger than other subgroups (*p* < 0.05, ANOVA followed by Dunett).

The study was conducted in two tertiary referral centers for balance disorders. The study received the approval of the local ethical research committee (CPP Est III) and from the French National Agency of Safety for Medicine and Health Products (number 2015-A00754-45). An informed consent was signed by all patients. All patients (but not controls) underwent a vestibular assessment with caloric and rotatory tests, videonystagmography analysis of gaze, pursuit, and saccade, and finally cervical vestibular evoked myogenic potentials (cVEMP). Eye movement analysis did not show signs of central involvement in this population.

BVP was defined according to the Barany Society criteria: the horizontal angular vestibulo ocular reflex (VOR) gain on both sides <0.6 (angular velocity 150–300°/s) and/or the sum of the maximal peak velocities of the slow phase caloric-induced nystagmus for stimulation with warm and cold water on each side <6°/s and/or the horizontal angular VOR gain <0.1 upon sinusoidal stimulation on a rotatory chair (0.1 Hz, *V*_max_ = 50°/s) and/or a phase lead >68 degrees with a time constant of <5 s (29). All BVP subjects had a hearing test to confirm their normal hearing. Control subjects were asymptomatic and not tested for hearing.

In UCI group, nine patients (60%) had a normal caloric test, five (33%) had a deficit in the same side as the implanted ear, and one (7%) had a bilateral deficit. Eleven (73%) UCI had bilateral normal responses to cVEMPs, four (27%) had a deficit the same side as the implanted ear.

In BCI group, caloric stimulations were obtained in five cases. There was a bilateral deficit in three cases (60%), a unilateral deficit in one case (20%), and a normal caloric test for one patient. Four (57%) BCI had bilateral normal responses to cVEMPs, two (29%) patients had a unilateral right deficit, and one (14%) had a bilateral deficit. No UCI or BCI patient corresponded to the criteria of BVP.

In the UCI group, eight patients were implanted on the right side and seven UCI on left side. In this group, 13 patients were implanted with Digisonic SP implant and Zebra Processor (Oticon Medical Inc., Vallauris, France), one patient with Hi-Res 90K implant and Naida processor (Advanced Bionics, Valencia, CA), and one with Nucleus Freedom implant and processor (Cochlear Inc., Lane Cove, Australia). Three patients had a hearing aid on the contralateral ear. UCI were implanted 6 ± 1.2 years before the inclusion. The pure-tone average threshold of the implanted ear was 39 ± 2.0 dB HL (*n* = 15). The aided pure-tone average threshold of the contralateral ear was 95 ± 6.2 dB HL (*n* = 15).

BCI were implanted 4 ± 1.3 years before test tests for the right ears and 2.8 ± 1.06 years for the left ears. The pure-tone average threshold was 43 ± 5.2 dB HL on the right and 44 ± 3.2 dB HL on the left. In this group, six patients were bilaterally implanted with Digisonic SP implant and Zebra Processor (Oticon Medical Inc., Vallauris, France), and one with Advanced Bionics Hi-Res 90K implant and Naida processor (Advanced Bionics, Valencia, CA).

All patients were evaluated on a dynamic posturography platform. Each test was conducted in silence, with a clockwise and counterclockwise rotating sound.

### Dynamic posturography

**Figure 1:**
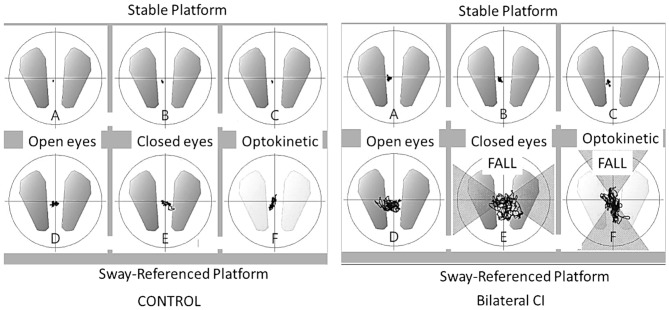
Center of pressure excursion surface (COPS). Ellipse containing 90% of all center of pressure positions (mm2) in six conditions on dynamic posturography for a control subject and a bilateral cochlear implant patient. The line shows the movement of pressure positions during the 30 s. acquisition and “FALL” indicates patient falls during the test.

The tests were performed on a conventional posturography plate bearing three pressure sensors (Balance Quest, Micromedical Technologies, Chatham, IL). The sampling rate was set at 50 Hz. The confidence ellipse surface containing 90% of all center of pressure positions was recorder and referred to as center of pressure excursion surface (COPS in mm2, Figure 1). A value of 300 was assigned to the test in case of fall. Sensory preferences and visual dependency indexes were calculated based on COPS measurements in different posturography conditions as follows (30): visual preference (eyes open, stable platform/eyes open, sway-referenced platform), vestibular preference (eyes open, stable/eyes closed, sway-referenced platform), proprioceptive preference (eyes open, stable/eyes closed, stable), visual dependency index (optokinetic stimulation, stable + optikenitic stimulation, sway-referenced)/(eyes closed, stable + eyes closed). Each condition was measured for 30 s and a 15-s break separated each condition.

In the two most unstable conditions (eyes closed sway-referenced, EC-SR and optikenitic stimulation, sway-referenced, OK-SR), wavelet and diffusion analyses were conducted: the energy consumption was measured in two axes (mediolateral: X and anterior-posterior: Y) in three frequency bands 0.05–0.5, 0.5–1.5, and 1.5–10 Hz by wavelet analysis. Based on this measure, a postural stability index (PSI) representing the total time during which no energy consumption was measured, and a postural control index (PCI) representing the total time with postural activity were automatically calculated by the software in the three frequency bands (Posturopro ® Software, Inserm, Marseille, France). A postural instability index (PII) deduced from the two latter parameters was studied (PII = PSI/PCI). The diffusion analysis estimated the extent of oscillation around an equilibrium state and its break-point by two additional parameters: the critical time (CT, in s) and the critical amplitude (CA, in mm^2^). Moreover, a Fractal analysis was conducted and the result was presented as the proportion of Hausdorff points (n/N) representing the percentage of stochastic position points among all sampled positions.

Subjects were stimulated by a rotating sound on the dynamic posturography platform in four trials (clockwise rotating sound, silence, anti-clockwise rotating sound, silence). The stimulus consisted of a rotating *Cocktail party* sound at 189°/s horizontally around the subject. The sound was delivered at 75 dB by a headphone (HD 205, Sennheiser, Wedemark, Germany) to mask the noise produced by the posturography platform. The rotating sound effect was created by CSoundQT® 3.1 Software (Pelican, Gumby Framework, New Haven, CT) based on head-relative transfer function.

In silence, control and BVP subjects were tested in a quiet room with the headphone off placed on ears. For UCI and BCI patients, the external processors and the hearing aids were removed.

### Statistics

Values were expressed as mean ± SD. Linear mixed models were used to access relationship between sound and gait of different subgroups. As the age could be a significant factor for the performances on the dynamic posturography, the model was corrected for age. A robust estimator of variance was used (31). Statistical tests were conducted on Stata (StataCorp LLC, College Station, TX).

## Results

### Posturography

#### Center of pressure excursion surfaces and sensory organization test

As expected, patients with bilateral vestibular loss had larger COPS than controls in eyes closed or optokinetic and sway-referenced conditions (Table 1, *p* < 0.05, Linear mixed models, *n* = 69). Interestingly, UCI and BCI had greater excursion surfaces than control subjects not only in sway-referenced conditions but also in eyes closed and stable platform condition (Table 1).

**Table 1:** Center of Pressure (COP) excursion surfaces as a function of posturography conditions in silence and rotating sound.

**Condition**	**Sound**	**Control**	**Unilateral CI**	**Bilateral CI**	**Bilat. vestibular loss**
		**(*n* = 37)**	**(*n* = 15)**	**(*n* = 7)**	**(*n* = 10)**
Eyes open stable	Silence	2.1 ± 2.74	3.3 ± 2.96	6.2 ± 8.21	2.4 ± 3.81
	CW	1.8 ± 1.86	3.6 ± 2.79	6.5 ± 5.92	3.5 ± 5.11
	CCW	1.6 ± 1.25	2.6 ± 1.47	5.0 ± 6.98	3.9 ± 5.74
Eyes closed stable	Silence	1.0 ± 0.86	**3.6** ± 4.79^*^	**4.1** ± 4.86^*^	32.8 ± 93.96
	CW	1.1 ± 0.94	**2.7** ± 2.05^*^	**2.8** ± 1.67^*^	6.9 ± 11.70
	CCW	1.3 ± 1.03	**3.5** ± 2.49^*^	**5.5** ± 5.80^*^	6.6 ± 11.37
Optokinetic stable	Silence	1.4 ± 1.68	6.5 ± 16.08	2.5 ± 1.66	32.1 ± 94.15
	CW	1.2 ± 1.15	22.2 ± 76.89	2.5 ± 2.03	34.0 ± 93.66
	CCW	2.3 ± 0.69	4.9 ± 1.63	3.7 ± 1.37	61.5 ± 39.75
Eyes open sway-ref.	Silence	7.0 ± 5.55	**59.8** ± 99.01^*^	**163.8** ± 130.21^*^	35.6 ± 93.18
	CW	6.4 ± 6.05	**39.2** ± 75.20^*^	**82.0** ± 99.10^*^	11.2 ± 18.51
	CCW	7.9 ± 7.89	**40.6** ± 74.57^*^	**110.9** ± 130.19^*^	14.0 ± 20.83
Eyes closed sway-ref.	Silence	18.4 ± 48.81	**220.0** ± 121.84^*^	**220.9** ± 135.29^*^	**139.3** ± 131.99^*^
	CW	26.2 ± 68.73	**126.8** ± 129.34^*^£	**258.9** ± 108.81^*^	**148.0** ± 136.39^*^£
	CCW	11.6 ± 21.86	**112.9** ± 119.85^*^£	**227.4** ± 124.00^*^	**180.9** ± 144.18^*^£
Optokinetic sway-ref.	Silence	35.2 ± 81.46	**135.5** ± 139.61^*^	**192.6** ± 134.74^*^	**129.1** ± 129.14^*^
	CW	27.0 ± 67.45	**94.6** ± 128.71^*^	**272.5** ± 72.87^*^£	**161.8** ± 132.78^*^
	CCW	20.6 ± 50.41	**101.3** ± 126.31^*^	**257.6** ± 109.61^*^£	**163.6** ± 140.44^*^

*Surfaces include 95% of all COPs and are expressed as mean ± SD in mm^2^. CW, Clockwise; CCW, Counter-clockwise; CI, Cochlear implant*.

* *p < 0.05 vs. control*.

*£p < 0.05 vs. silence in the same condition and group, linear mixed model corrected for age, n = 69*.

*Bold values represent significant differences of the parameter in comparison to control or versus silence*.

Rotating sound seemed to influence COPS differently in patients with bilateral vestibular loss and in those with UCI:COPS increased with sound in the bilateral vestibular loss group (*n* = 10) in EC-SR condition, indicating a destabilizing effect, while it decreased in UCI in the same environment suggesting stabilization (*n* = 15, *p* < 0.05, linear mixed model corrected for age, *n* = 69, Table 1).

In the optokinetic and sway-referenced condition, BCI had also larger COPS with rotating sounds (CW and CCW) than in silence suggesting a destabilizing effect (*p* < 0.05, linear mixed model corrected for age, *n* = 69, Table 1) while other groups did not seem to be influenced in this condition.

SOT in silence revealed a lower proprioceptive preference index in subjects with bilateral vestibular loss than in controls (Table 2). In contrast, UCI patients had a lower vestibular preference index than controls. In BCI subjects, both vestibular and visual preference indexes were lower than controls (Table 2, linear mixed models corrected for age, *n* = 69). Sound did not seem to influence sensory preferences (Table 2).

**Table 2:** Sensory preference index based on center of pressure excursion surfaces in silence and rotating sound.

**Sensory preferences**	**Sound**	**Control**	**Unilateral CI**	**Bilateral CI**	**Bilat. vestibular loss**
		**(*n* = 37)**	**(*n* = 15)**	**(*n* = 7)**	**(*n* = 10)**
Visual	Silence	0.4 ± 0.66	0.2 ± 0.16	**0.1** ± 0.10^*^	0.3 ± 0.23
	CW	0.5 ± 0.61	0.4 ± 0.53	**0.2** ± 0.15^*^	0.8 ± 1.24
	CCW	0.5 ± 1.31	0.3 ± 0.50	**0.1** ± 0.10^*^	0.4 ± 0.32
Vestibular	Silence	0.5 ± 1.39	**0.04** ± 0.07^*^	**0.1** ± 0.07^*^	0.2 ± 0.38
	CW	0.6 ± 1.37	**0.5** ± 1.42^*^	**0.1** ± 0.11^*^	0.1 ± 0.22
	CCW	0.7 ± 2.64	**0.1** ± 0.23^*^	**0.1** ± 0.10^*^	0.2 ± 0.45
Proprioceptive	Silence	2.3 ± 3.7	1.6 ± 1.12	2.1 ± 2.79	**0.8** ± 0.76^*^
	CW	2.0 ± 2.02	1.9 ± 1.97	2.0 ± 0.96	**1.0** ± 1.00^*^
	CCW	1.6 ± 1.38	0.9 ± 0.54	1.3 ± 0.75	**1.2** ± 0.92^*^
Visual dependency	Silence	3.0 ± 6.74	1.0 ± 1.86	1.1 ± 0.50	20.0 ± 59.21
	CW	1.4 ± 1.51	2.1 ± 6.00	3.7 ± 7.35	3.2 ± 6.92
	CCW	2.4 ± 4.11	1.4 ± 2.07	1.3 ± 0.82	1.4 ± 1.04

*Values are expressed as mean ± SD. CW, Clockwise; CCW, Counter-clockwise; CI, Cochlear implant*.

* *p < 0.05 vs. control, linear mixed model corrected for age, n = 69*.

*Bold values represent significant differences of the parameter in comparison to control or versus silence*.

Although CIs had a COPS similar to controls in the easy condition (eyes open, stable platform), they showed greater COPS in difficult conditions (closed eyes, sway referenced), together with a reduced vestibular preference. This suggested a compensated vestibular deficit in accordance with caloric and otolithic tests.

### Wavelet, diffusion, and fractal analysis of stabilometry

In silence and in EC-SR condition, PII was higher in UCI than in control suggesting more instability (Table 3, *p* < 0.05, Linear mixed models, *n* = 69). This difference was not observed in OK-SR condition. Other groups of patients had similar PII to the control in EC-SR and OK-SR conditions (Table 3, *p* < 0.05, linear mixed models corrected for age, *n* = 69). Sound did not seem to influence PII (Table 3).

**Table 3:** Postural instability index.

**Postural instability index**	**Sound**	**Control**	**Unilateral CI**	**Bilateral CI**	**Bilat. vestibular loss**
		**(*n* = 37)**	**(*n* = 15)**	**(*n* = 7)**	**(*n* = 10)**
Eyes closed sway-ref.	Silence	3.2 ± 0.96	**4.6** ± 2.06^*^	3.9 ± 2.80	4.0 ± 2.46
	CW	3.1 ± 1.21	**4.9** ± 0.97^*^	4.6 ± 2.22	4.4 ± 2.22
	CCW	2.9 ± 0.98	**4.8** ± 1.07^*^	4.7 ± 2.24	4.4 ± 2.31
Optokinetic sway-ref.	Silence	3.4 ± 1.22	4.1 ± 1.35	3.7 ± 2.59	4.7 ± 1.78
	CW	3.3 ± 1.13	3.7 ± 1.73	4.4 ± 2.31	5.4 ± 1.51
	CCW	3.3 ± 1.19	4.2 ± 1.20	4.5 ± 2.26	4.4 ± 2.21

*Values are expressed as mean ± SD. CW, Clockwise; CCW, Counter-clockwise; CI, Cochlear implant*.

* *p < 0.05 vs. control, linear mixed model, corrected for age n = 69*.

*Bold values represent significant differences of the parameter in comparison to control or versus silence*.

Diffusion analysis revealed higher CAs in BVP, UCI, and BCI patients than control in EC-SR and OK-SR conditions regardless of sound conditions (*p* < 0.05, linear mixed models corrected for age, *N* = 69, Table 4). Critical time did not differ between groups (linear mixed models corrected for age, *n* = 69, Table 5).

**Table 4:** Critical time.

**Critical time**	**Sound**	**Control**	**Unilateral CI**	**Bilateral CI**	**Bilateral vestibular loss**
		**(*n* = 37)**	**(*n* = 15)**	**(*n* = 7)**	**(*n* = 10)**
Eyes closed sway-ref.	Silence	0.8 ± 0.35	1.0 ± 0.81	0.6 ± 0.52	1.0 ± 0.72
	CW	0.9 ± 0.37	1.2 ± 0.56	1.0 ± 0.60	1.0 ± 0.50
	CCW	1.0 ± 0.59	1.0 ± 0.55	0.8 ± 0.44	1.0 ± 0.65
Optokinetic sway-ref.	Silence	1.1 ± 1.31	1.1 ± 0.50	0.9 ± 0.82	0.8 ± 0.64
	CW	1.1 ± 0.87	0.9 ± 0.61	0.9 ± 0.88	0.8 ± 0.43
	CCW	1.1 ± 0.71	1.0 ± 0.38	0.7 ± 0.35	0.9 ± 0.53

*This parameter was estimated by diffusion analysis of stabilometry during dynamic posturography. Values are expressed as mean ± SD in seconds. CW, Clockwise; CCW, Counter-clockwise; CI, Cochlear implant. The was no difference between subgroups or experimental conditions (linear mixed model corrected for age, n = 69)*.

**Table 5:** Critical amplitude.

**Critical amplitude**	**Sound**	**Control**	**Unilateral CI**	**Bilateral CI**	**Bilat. vestibular loss**
		**(*n* = 37)**	**(*n* = 15)**	**(*n* = 7)**	**(*n* = 10)**
Eyes closed sway-ref.	Silence	350.9 ± 674.64	**2,787.5** ± 2,510.37^*^	**1,938.2** ± 2,782.11^*^	**2,608.9** ± 2,886.14^*^
	CW	456.2 ± 1,020.90	**2,429.0** ± **2,909.74** ^*^	**2,457.6** ± 2,240.32^*^	**2,401.5** ± 2,118.99^*^
	CCW	373.2 ± 1,029.16	**2,352.7** ± 3,186.51^*^	**2,509.7** ± 2,462.20^*^	**2,764.5** ± 2,601.43^*^
Optokinetic sway-ref.	Silence	385.5 ± 606.4	**1,220.4** ± 1,619.2^*^	**1,135.2** ± 1,005.7^*^	**1,920.1** ± 1,680.2^*^
	CW	456.2 ± 1,020.9	**2,429.0** ± 2,909.7^*^	**2,457.6** ± 2,240.3^*^	**2,161.3** ± 2,137.3^*^
	CCW	360.4 ± 681.4	**2,787.5** ± 2,510.4^*^	**1,938.2** ± 2,782.1^*^	**2,608.9** ± 2,886.1^*^

*This parameter was estimated by diffusion analysis of stabilometry during dynamic posturography. Values are expressed as mean ± SD (mm^2^). CW, Clockwise; CCW, Counter-clockwise; CI, Cochlear implant*.

* *p < 0.05 vs. control, linear mixed model corrected for age, n = 69*.

*Bold values represent significant differences of the parameter in comparison to control or versus silence*.

Fractal analysis showed lower proportion of Hausdorff points in UCI and BCI subjects than in controls in Y (roll) axis in EC-SR and OK-SR conditions (*p* < 0.05, linear mixed models corrected for age, *n* = 69, Table 6). Sound did not seem to influence the proportion of Hausdorff points in the Y axis in any subgroup (Table 6). No difference between subgroups or effect of sound could be observed in the X axis (pitch, data not shown).

**Table 6:** Proportion of Hausdorff points in Y (Roll) axis.

**Exp. condition**	**Sound**	**Control**	**Unilateral CI**	**Bilateral CI**	**Bilateral vestibular loss**
		**(*n* = 37)**	**(*n* = 15)**	**(*n* = 7)**	**(*n* = 10)**
Eyes closed sway-ref.	Silence	1.6 ± 1.27	**0.6** ± 0.77^*^	**0.4** ± 0.43^*^	1.7 ± 1.08
	CW	1.7 ± 1.75	**0.7** ± 0.81^*^	**0.7** ± 0.51^*^	1.7 ± 1.17
	CCW	1.7 ± 2.03	**0.9** ± 0.88^*^	**0.9** ± 0.96^*^	1.2 ± 0.86
Optokinetic sway-ref.	Silence	1.6 ± 1.42	**0.9** ± 0.83^*^	**0.3** ± 0.24^*^	1.8 ± 1.04
	CW	1.5 ± 1.34	**0.6** ± 0.45^*^	**0.5** ± 0.31^*^	1.7 ± 1.49
	CCW	1.5 ± 2.41	**0.7** ± 0.89^*^	**0.5** ± 0.37^*^	1.3 ± 0.84

*This parameter was calculated by fractal analysis of dynamic stabilometry. Values are expressed as mean ± SD. CW, Clockwise; CCW, Counter-clockwise; CI, Cochlear implant*.

* *p < 0.05 vs. control, linear mixed model corrected for age, n = 69*.

*Bold values represent significant differences of the parameter in comparison to control or versus silence*.

## Discussion

In this study, we showed that the hearing afferences could have an impact on the gait especially when other sensory inputs are impaired. As expected, patients with BVP, UCI, and BCI had poorer posturography performances than the control and their sensory organization was altered. Interestingly, the rotating sound reduced the COPS in patients with UCI and no stereophony but increased COPS in patients with BVP enjoying stereophony and BCI with binaural hearing in sway-referenced conditions and disturbed visual input. In contrast, control subjects did not modify their COPS under the sound effect suggesting a different hierarchy of sensory inputs in these individuals. The destabilizing effect of the rotating sound in BVL and BCI patients could be enhanced by the vestibular deficit in these subjects. This deficit might have modified the hierarchy of sensory inputs for balance.

Two limitations of our study were that the mean age of the control population was lower than other subgroups, and that although control subjects were totally asymptomatic, they were not explored by audiometry. We corrected the effect of age in our multivariate analysis, but these two aspects might limit the comparison between subgroups.

Several studies have already reported the effect of the sound on the gait or vestibular function with contradicting results: Some studies reported higher stability (9, 11–13, 32), while others described poorer balance performances (10, 21, 22). The apparent contradiction could lie in the nature of the stimulus: stable vs. moving source.

Stevens et al. (32) tested the impact of the sound on the gait by dynamic posturography in 12 control and six patients with neurological diseases. They delivered a stable white noise via four earth-referenced speakers placed around the subject during the six conditions of posturography and showed that this type of stimuli decreased the COPS in both patients and controls. By comparing head-fixed stationary sound to silence, the authors did not find an improvement of the gait and concluded that the effect of an earth-fixed sound is probably based on localization cues more than on alertness. This observation was in accordance with a previous study in which stationary music delivered by a headphone did not influence the gait during posturography (26). Moreover, the stabilizing effect of earth-fixed stationary sounds were in accordance with other studies in healthy subjects evaluating gait by the Fukuda stepping test (9) and Romberg test (12).

In addition to providing cues on the distance and orientation of the body relative to earth-referenced sound sources, recent reports suggest that stable sound sources may interact with the visual input (33). Indeed, directing the gaze toward the source without moving the head significantly enhances the detection of interaural time and intensity differences (33). This observation indicates a visual and auditory interaction possibly at the brainstem level which could also benefit to the gait.

The effect of moving sound fields has also been investigated by stabilometry in healthy subjects but with contradictory results. While some authors report that moving sounds appear to increase sway (1, 21, 22), others observe a stabilizing effect of rotatory sounds and argue that contrary to moving visual cues, mobile sound sources are easier to identify and consequently more valuable in a multisensory processing of the balance (27).

The type of sound used in the experimental protocol might also explain the discrepancies between the reports on this subject (1, 10, 34). In 1960, Hennebert stated that rotating continuous pure tone elicited little vestibular response (nystagmus, Romberg test) while discontinuous pure tones with regular interruptions at a 3–5 Hz frequency have a higher impact. Similarly, white noise or complex sounds such as music yielded better responses (1). In this report, no quantification of the effects and quantitative comparison was provided. To our knowledge, no other study has compared the effect different sounds on the gait. For our subjects, we chose a cocktail party noise in order to be realistic and close to daily-life situations.

In our study, the rotating sound algorithm provided an impression of source displacement to patients with binaural hearing (BVP and BCI) but appeared as a sound oscillating in amplitude in the implanted ear of UCI patients. Based on previous reports, this could explain the destabilizing effect of the rotating sound in BVP and BCI subjects in contrast to the stabilizing impact on the UCI patients. Our sound stimuli did not disturb the healthy subjects even in conditions where the visual and proprioceptive inputs were hampered. This observation suggests that hearing afferences have a more prominent effect on the posture when other afferences (especially vestibular) are damaged.

The impact of CI on the vestibular system is a concern among otologists, mainly because the surgical trauma of the implantation in an already fragile inner ear may partly destroy the vestibule. This possibility influences the rehabilitation strategy especially in case of bilateral implantation (35). However, few studies have focused on the potential advantage of auditory input on the gait as a result of a richer multisensory input (14, 36, 37). While the effect of an active CI is undetectable by dynamic posturography if the patient is not stimulated by sound (37), patients seem to performed better with their CI on and an earth-referenced white noise in dark in comparison to the same situation with deactivated CI (14). Similarly, patients with hearing aids appear to benefit from the enhanced auditory input for their balance performance in the presence of a stationary sound (36).

Observations on dynamic posturography are also in accordance with several publications reporting an increase in risk of falls beginning in mild hearing losses and proportional to its severity: 1.4 X for every 10 dB loss in senior population (12, 13, 38–40). Additionally, the idea that auditory input can be readily integrated in the multisensory gait control is also supported by the observation that translating hip and trunk movement into sound at delivering it to the subjects through headphone (auditory biofeedback) enhanced postural control in both BVP and healthy subjects (11).

Wavelet, diffusion, and fractal analysis of stabilometry in dynamic posturography have already been reported as meaningful indicators of balance performance and efficiency (41–43). More than instability, they are indicators of energy consumption and balance control strategy: strict correction of COP displacements requiring more energy vs. a more tolerant strategy (44). Wavelet and diffusion analysis, confirmed deductions from COPS showing lower balance performances in patients with CI and in BVP compared to controls. However, they did not indicate a significant modification of energy consumption or balance strategy under the rotating sound effect. This could be explained by large interindividual variations (especially for the CA) and the fact that dynamic posturography evaluates the balance in a standing position and not during movements (walking). Indeed, walking is probably a more ecological manner to evaluate the multisensory integration in balance (45).

Interaction between auditory and vestibular information seems to take place at the cortical level. The temporo–parietal junction, connecting the auditory, somatosensory, and visual cortices is involved in a multimodal representation of space. It occupies the posterior portion of the superior temporal plane and the superior temporal gyrus. It contains trimodal neurons with receptive fields over the head–neck–shoulder region potentially involved in head orientation (46). A recent fMRI investigation suggests that superior temporal gyrus (planum temporale) and the posterior insula are particularly involved in the processing of both auditory and vestibular information (47). Sound–movement interaction may also be processed at a subcortical level. Recent studies on the influence of gaze direction on the auditory spatial resolution suggest a multisensory integration in the brainstem, presumably in the superior colliculus (32).

In conclusion, rotating sound influences the gait and alters the balance strategy in patients with CI and in BVP. While it destabilizes patients with binaural hearing (BCI and BVP), it seems to stabilize those with monaural hearing (UCI). These observations indicate the integration of binaural auditory cues for the balance control in patients with BCI.

## Data availability statement

The raw data supporting the conclusions of this manuscript will be made available by the authors, without undue reservation, to any qualified researcher.

## Author contributions

AB, MT, and CG designed the study. BD, CG, MT, and AB developed the experimental setup. CG, BD, and SH evaluated the subjects. CG, SA, and AB analyzed the data and prepared the manuscript.

### Conflict of interest statement

The authors declare that the research was conducted in the absence of any commercial or financial relationships that could be construed as a potential conflict of interest.
